# Transcriptional circuitry atlas of genetic diverse unstimulated murine and human macrophages define disparity in population-wide innate immunity

**DOI:** 10.1038/s41598-021-86742-w

**Published:** 2021-04-01

**Authors:** Bharat Mishra, Mohammad Athar, M. Shahid Mukhtar

**Affiliations:** 1 Department of Biology, University of Alabama At Birmingham, 464 Campbell Hall, 1300 University Boulevard, Alabama, 35294 USA; 2 UAB Research Center of Excellence in Arsenicals, Department of Dermatology, School of Medicine, University of Alabama At Birmingham, Alabama, 35294 USA; 3 Nutrition Obesity Research Center, University of Alabama At Birmingham, 1675 University Blvd, Birmingham, AL 35294 USA; 4 Department of Surgery, University of Alabama At Birmingham, 1808 7th Ave S, Birmingham, AL 35294 USA

**Keywords:** Computational biology and bioinformatics, Genetics, Systems biology

## Abstract

Macrophages are ubiquitous custodians of tissues, which play decisive role in maintaining cellular homeostasis through regulatory immune responses. Within tissues, macrophage exhibit extremely heterogeneous population with varying functions orchestrated through regulatory response, which can be further exacerbated in diverse genetic backgrounds. Gene regulatory networks (GRNs) offer comprehensive understanding of cellular regulatory behavior by unfolding the transcription factors (TFs) and regulated target genes. RNA-Seq coupled with ATAC-Seq has revolutionized the regulome landscape influenced by gene expression modeling. Here, we employ an integrative multi-omics systems biology-based analysis and generated GRNs derived from the unstimulated bone marrow-derived macrophages of five inbred genetically defined murine strains, which are reported to be linked with most of the population-wide human genetic variants. Our probabilistic modeling of a basal hemostasis pan regulatory repertoire in diverse macrophages discovered 96 TFs targeting 6279 genes representing 468,291 interactions across five inbred murine strains. Subsequently, we identify core and distinctive GRN sub-networks in unstimulated macrophages to describe the system-wide conservation and dissimilarities, respectively across five murine strains. Our study concludes that discrepancies in unstimulated macrophage-specific regulatory networks not only drives the basal functional plasticity within genetic backgrounds, additionally aid in understanding the complexity of racial disparity among the human population during stress.

## Introduction

Macrophages are functionally diverse and conserved cells across tissues in the mammalian hematopoietic system. They have been implicated in versatile biological processes including tissue homeostasis, immunity, development, disease and tissue repair^1^. The differences in anatomy, transcriptome expression and functional pathways of tissue macrophage cells are critical to maintaining homeostasis in all tissues^2^. Therefore, a study on macrophages provides a highly selective mechanism underlines homeostatic and regulatory immunity^3^. However, these strategies become more complex when we investigate the macrophage regulatory functions in a diverse population of genetic strains/variants^4^. The genetic regulatory circuits have a significant role in determining distinct transcriptional rewiring to maintain homeostasis and immunity across genetic diversity^5^. These macrophage-specific regulatory networks are assembled by transcription factors (TFs) specific promoters and enhancers elements determining their lineage or enforce tissue-limiting properties^6^. The differentiation in regulatory circuits determines the plasticity in macrophage associated genetic crosstalk between metabolic pathways and regulation of gene expression^7^. Several genetic variants within regulatory regions of the genome have also been reported for disease and other traits association illustrating the effect of TFs in gene regulation^8–10^. These genetic variations and alterations of TF binding motifs are the underlying mechanism(s) for the regulation of gene expression and biological function^11,12^. Consistent with this, several inbred murine models have been developed over the years to understand the basis of genetic variations representing phenotype^5^. Nevertheless, five murine models are shown to exemplify almost all genomic diversity associated with human genetic variations^4^. These five murine models; C57BL/6J (C57), BALB/cJ (BALB), NOD/ShiLtJ (NOD), PWK/PhJ (PWK), and SPRET/EiJ (SPRET) mice represent approximately > 50 million SNPs + InDels of genes associated with a difference amongst two individual human beings^13,14^.

With the advent of novel omics tools and platforms in the last two decades, a large number of genome-scale datasets have been accumulated in the biological sciences research community^15^. The fundamental challenge, however, remains on how to handle and analyze huge and multidimensional data sets including genomes, transcriptomes, proteomes, metabolomes, and regulomes. Towards this, the integrative systems biology analyses have been effectively applied to investigate multidimensional data sets individually and compiled collectively to understand the system-wide complexity^16^. Systems biology has transpired as an efficient practice with the remarkable advancement in network integration techniques to decode the biological genetic intricacy^17^. Networks encompass a set of systems components (nodes; genes/proteins or their products) interactions (edges) among themselves to generate a cellular response^18^. These interactions specifically, gene co-expression and protein-DNA interaction, facilitate a functional rewiring to any perturbation in the cellular processes^19^. Gene co-expression network construction and analyses have been applied to identify the significant players/modules in the biological system through network architectural analyses^20,21^. While protein-DNA interaction networks are utilized to establish gene functional pathway regulation^22^. Typically, biological networks display scale-free topology; few nodes retaining heightened interactions, defined by power-law distribution^23–26^. Network topology measures are physical or structural characteristics of the network, critical for deciphering the structural properties (centralities) to reveal novel components of biological network ^17,27^. Some of the highly used biological network centralities are degree, betweenness centrality, shortest path length, and cluster coefficient^17,24,28^. Indeed, it has been established that high degree (hubs) and high betweenness centrality (bottlenecks) nodes of a network are significantly crucial players in most of the biological processes^21,29,30^. Provided, plentiful biological systems exhibit analogous network formation and topology, centralities feature within a network may unravel the indicators of significant conditional nodes^17,18,31^.

**Figure 1:**
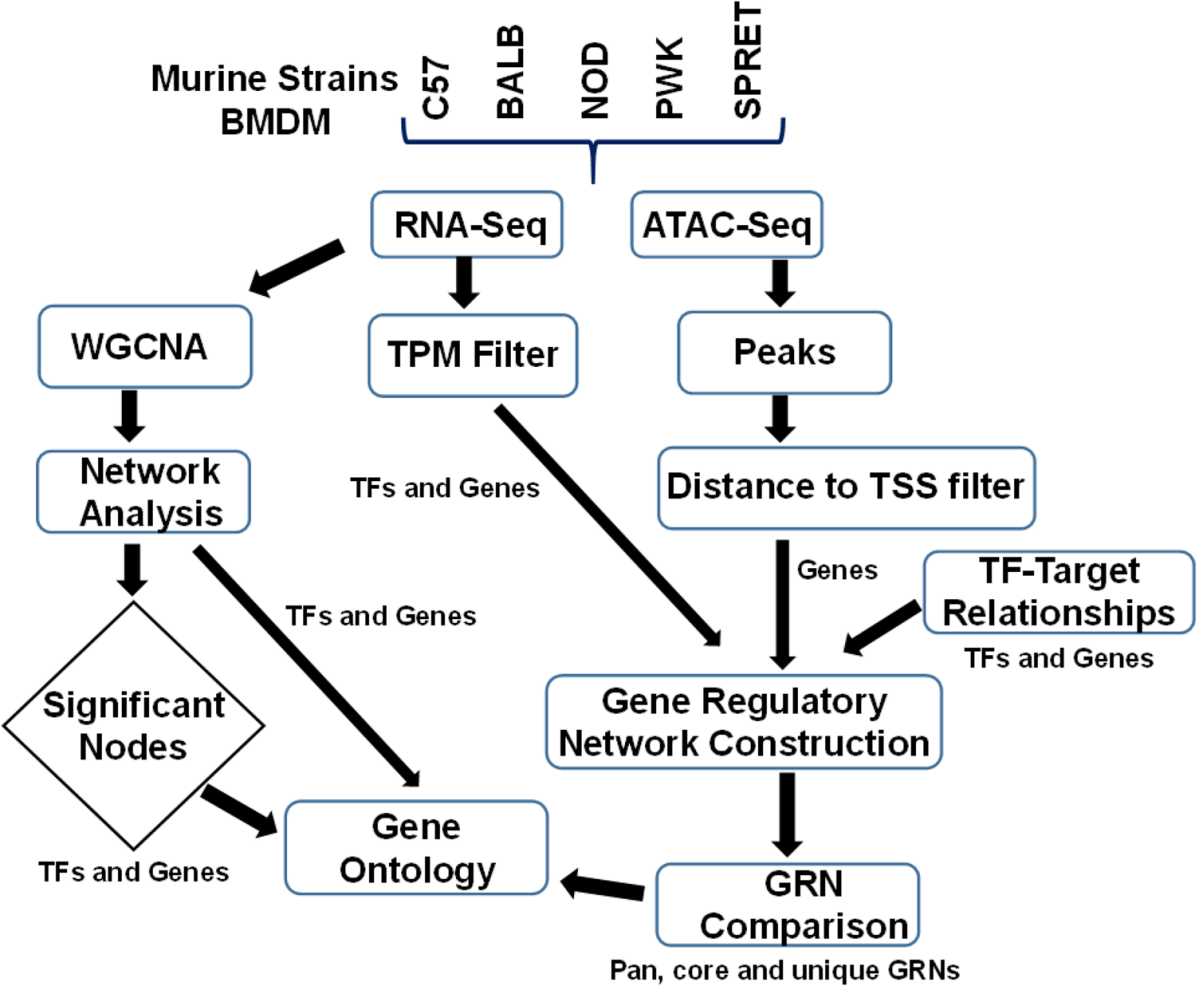
Integrative multi-omics pipeline to model the unstimulated bone marrow-derived macrophage (BMDM) putative gene regulatory networks (GRNs) in five genetically defined murine strains.

Protein-DNA interactions, specifically gene regulatory networks (GRNs) orchestrate the genotypic functional diversity by synchronizing the regulation of functional pathways^4,32–38^. GRNs are collections of transcription factor (TF) footprints on gene’s promoter region to govern (activate/inhibit) the gene expression, reconstructed during specialized biological fitness. TFs motif bind to the promoter region of genes with open chromatin to regulated gene expression^39^. Recent advances in genome-scale chromatin accessibility measurement techniques; the Assay for Transposase-Accessible Chromatin (ATAC)-seq has overcome the limitations of typical techniques (ChIP-seq and FAIRE-seq) and is a widely recognized tool^40^. The chromatin accessibility measurement based on TF binding provides the putative TF-gene interactions, enhanced by gene expression (RNA-seq) and prior TF-target gene interactions^41^. GRNs are bipartite graphs, provide exceptional illustrations of genetic scale-free network topology^23,42^. Also, GRNs are directed graphs with edge polarity; TF binding to regulatory regions of a gene/other TF to govern gene/other TF expression, but not the other way round. Individual phenotypic plasticity is quantitatively driven through the underlying system-wide GRNs^36^. Consequently, modeling and structural investigation of GRNs delivers a tremendous prospect to unravel the regulatory mechanism driven unstimulated macrophage phenotypic plasticity. Multiple studies have reported the genome-wide chromatin accessible remodeling signatures in LPS, environmental and other induced macrophages^4,43–45^. However, the basic differences amongst the basal GRNs of unstimulated bone marrow macrophages are not explored in detail of genetically diverse murine strains, which could be crucial to understand the stimulatory and plastic behavior of these immune cells in order to tackle external stimuli. Here, we explore GRN differences that might be associated with the variable response towards any macrophage associated disease signatures in global population demographics. We modeled five genetically diverse basal homeostasis GRNs based on unstimulated macrophage ATAC-seq and RNA-seq and identify conserved and distinct GRN components participate in biological functions during basal homeostasis (Fig. 1)^4^. Additionally, we generated bone marrow-derived macrophages (BMDMs) associated basal homeostatic gene co-expression network form transcriptome of five murine strains to discover significant topological and biological functional identities and players (TFs and genes) regulating basal homeostasis during unstimulated macrophages. Furthermore, we used integrative multi-omics methods to model the regulome atlas to determine the gene regulatory relationship to maintain innate immunity and basal homeostasis. As a result, our integrative approach helped us to unravel the conserved as well as discrete regulatory networks in five genetically diverse murine strains with crucial implications in maintaining immunity and basal homeostasis. Moreover, we also compared inflammatory and type 1 IFN response marker genes expression behavior in different human races and five murine strains. Taken together, our findings establish the effectiveness of a consolidative network-centric approach in predicting physical properties of strain-specific GRNs, with significant implications in the interpretation of regulatory repertoire complexity driven phenotypic plasticity.

## Results

### Shared and distinct expression of resting macrophage to maintain basal homeostasis in genetically diverse mice strains

**Figure 2:**
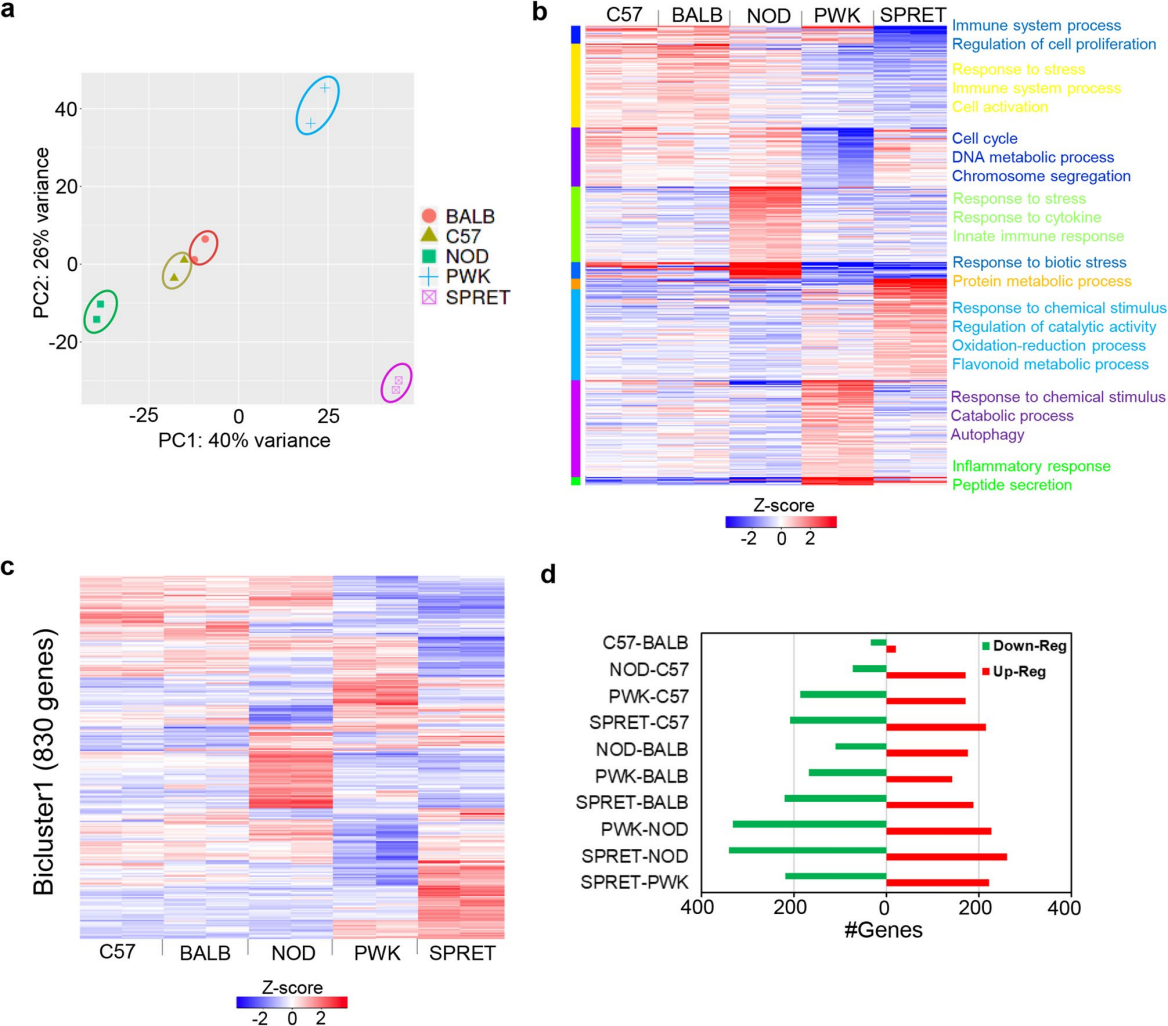
Unified and distinct gene expression patterns of unstimulated bone marrow-derived macrophage (BMDM) across diverse murine strains reveal significant diversified immune system response signal pathways to maintain basal hemostasis. (**a**) PCA visualization of the RNA-seq in five mice strains (C57, BALB, NOD, PWK, and SPRET) was labeled with distinct colors. The plot showing highly consistent RNA-seq clustered together for each mice strain. (**b**) Heatmap of 6000 most variable expressed genes out of 12,218 BMDM expressed genes in five mice strains based on *k*-means clustering along with significantly enriched biological processes. The clustered columns represent the expression in mice strains, whereas each row represents a gene (adjusted *P*-value < 0.05, expression colored based on TPM z-score). (**c**) CC Bicluster algorithm (BCCC) discovered 830 highly correlated genes in cluster1 and significantly enriched in immune systems biological process across five mice strains (colored based on TPM z-score). (**d**) The number of differential expressed genes (DEGs) maintaining basal homeostasis between different mouse strains (*FDR* = 0.05, FC ≥ |2|). Red represents up-regulated and the green represents down-regulated genes from one to another strain. C57 and BALB have the least number of DEGs.

To investigate the gene expression behavior of unstimulated macrophages, we utilized five genetically defined murine strains bone marrow-derived macrophage (BMDM) transcriptomics expression data^4^. The principal component analysis (PCA) of normalized read counts data revealed the variation of macrophage transcriptome amid five murine strains (C57, BALB, NOD, PWK and SPRET) (Fig. 2a, Supplementary Table S1). Closely clustered groups are strains that account for approximately 40% of the observed variation within the first two principal components. Strikingly, the analysis illustrates that C57 and BALB transcriptome are closer to each other than any other understudied mice strain transcriptome (NOD, PWK and SPRET). However, the gene expression cluster pattern analysis replicated the relationships shown by PCA as well as revealed that most of the genes follow the same expression behavior, whereas some genes are distinct to certain mouse strains. To examine the gene clustering behavior, we performed *k*-means clustering analysis that demonstrates that 6000 out of 12,218 BMDM most variable genes are clustered in nine clusters and enriched in significant biological processes in five mice strains (adjusted *P*-value < 0.05) (Fig. 2b, Supplementary Table S1). Interestingly, the first three clusters enriched in the immune system process, regulation of cell proliferation, response to stress, cell activation, cell cycle, and DNA metabolic process displays most genes are expressed higher in C57, BALB and NOD strains than PWK and SPRET. While three distinct clusters with higher expressed genes in NOD, SPRET and PWK strains are enriched in response to cytokine, oxidation–reduction process, flavonoid metabolic process, catabolic process, autophagy, and peptide secretion (adjusted *P*-value < 0.05) (Fig. 2b, Supplementary Table S1). Consistently, bicluster cluster coefficient algorithm (BCCC)^46^ identified 820 highly co-regulated genes significantly enriched in immune systems process and display both shared and distinct gene expression across five mice strains (Fig. 2c, Supplementary Table S1). Particularly, the efficient node-deletion algorithm identified differences in NOD, PWK and SPRET strains immune gene transcriptome expression. To identify the significant gene expression variations amongst five murine strains at unstimulated macrophage, we performed differential gene expression analysis (*FDR* < 0.05, log2FC >|2|). Interestingly, strain pairwise comparison in BMDM transcriptome uncovered > 400 differential expressed genes (DEGs) between SPRET-NOD, PWK-NOD, SPRET-PWK, SPRET-C57, and SPRET-BALB at resting macrophage (*FDR* < 0.05, log2FC >|2|) (Fig. 2d, Supplementary Table S1). While three pairwise comparisons between NOD-BALB, NOD-C57, C57-BALB uncovered < 300 DEGs at resting macrophage. Remarkably, we identified C57-BALB pair with least (55) enriched in antigen processing and presentation, humoral immune response, and SRP-dependent co-translational protein targeting to the membrane. Whereas, SPRET-NOD pair with most (602) DEGs enriched in interferon signaling, neutrophil degranulation, regulation of cytokine production, leukocyte migration, response to type I interferon, regulation of MAPK cascade, leukocyte proliferation during unstimulated macrophage. Given the aforementioned inter-strain similarities and differences of gene expression are responsible for the multitude of the intensity of immune responses towards multiple external stresses^4,5^.

### TFs and immunity-related genes are enriched in highly connected and correlated modules

**Figure 3:**
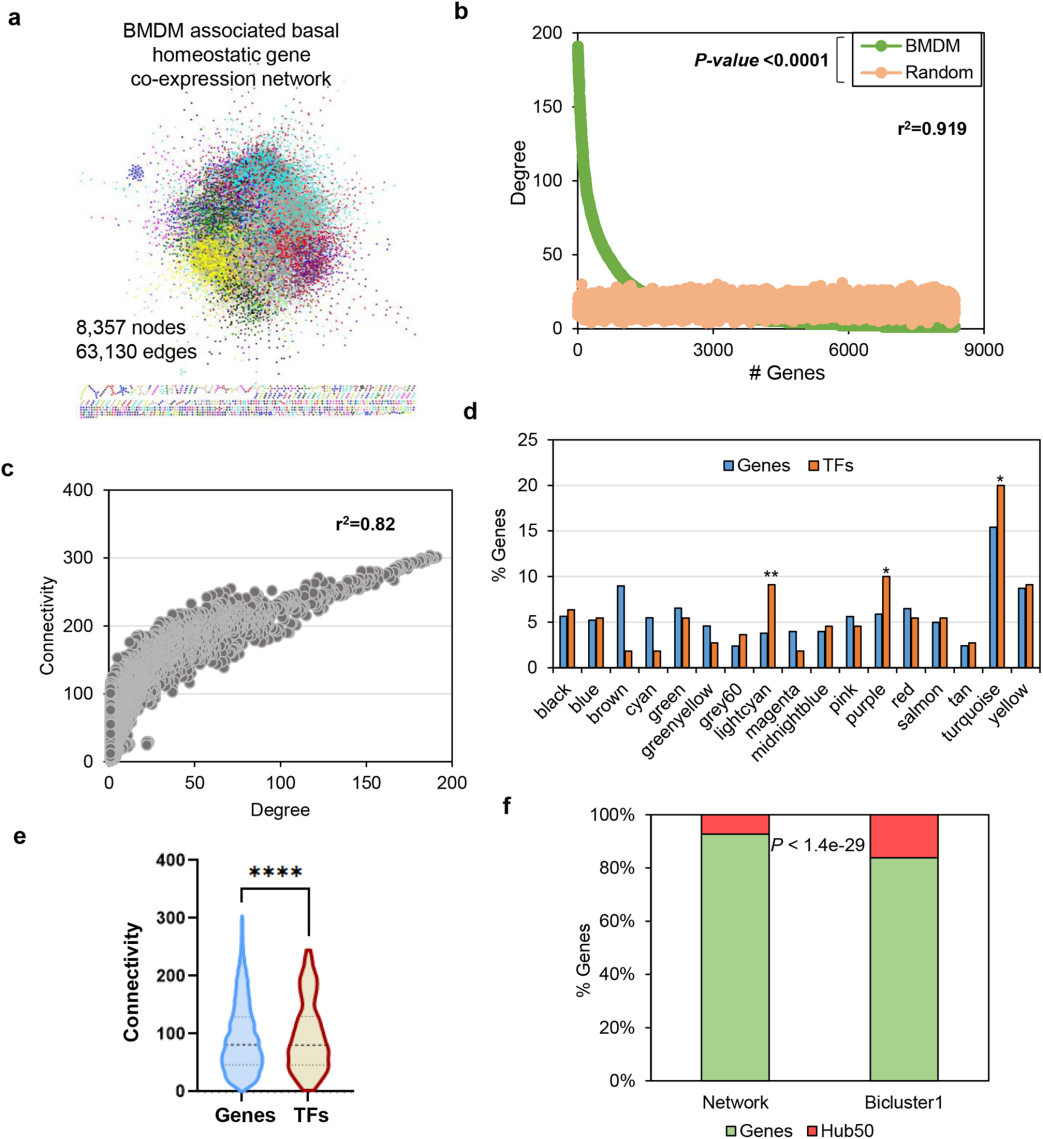
Transcription factors (TFs) and immunity genes are enriched in the co-expression network of unstimulated macrophage. (**a**) BMDM associated basal homeostatic gene co-expression network (8357 nodes and 63,130 edges) with 17 significant modules. (**b**) The degree distribution of nodes in BMDM associated homeostatic gene co-expression network (*r*^2^ = 0.92, Mann Whitney test *P* < 0.0001) and random network. Only 7.86% (657) of nodes have a high degree (Hub^50^) representing the scale-free property of the co-expression network. (**c**) The relationship between connectivity and degree of nodes shows a significant positive correlation (r^2^ = 0.82) suggesting the Hub^50^ nodes and their modules are highly connected. (**d**) The distribution of genes and TFs (in percent) in represented modules with turquoise having a maximum 1205 (15.4%) genes and 22 TFs (20%, Hypergeometric enrichment test *P* < 0.05), and grey60 having minimum 186 genes (2.3%) and 4 TFs (3.63%) of all co-expressed genes/TFs. Also, TFs in purple and lightcyan modules are significantly enriched than genes assigned these modules (Hypergeometric test *P* < 0.01 and 0.05, respectively). (**e**) The average connectivity of all TFs is significantly higher (96.09) than the genes (93.02) in the co-expression network (Wilcoxon matched-pairs signed rank test *P*-value < 0.0001). (**f**) Immune-related genes form bicluster1 (Fig. 1e) are significantly enriched in Hub^50^ (Hypergeometric enrichment test, *P* < 1.4e−29) representing their importance in the co-expression network.

To study the correlation between TFs and immunity-related genes in different mice strains, we performed co-expression network analysis by WGCNA^47^. This lets us identify a BMDM associated homeostatic gene co-expression network containing 17 significant modules and one insignificant module (grey) with 8357 nodes (genes) and 63,130 edges (pairs/connections) (Supplementary Fig. S1a, Fig. 3a, Supplementary Table S2). Among them, turquoise is the largest module with 1227 highly clustered genes enriched in the cellular protein catabolic process, organelle assembly, membrane trafficking, autophagy, positive regulation of neurogenesis, and neutrophil degranulation. While, grey60 is the smallest modules with 190 clustered genes enriched in regulation of ion transport, adaptive immune system, regulation of lipid metabolic process, gland development, and organelle biogenesis and maintenance. Noticeably, grey is the insignificant module with the least clustering coefficient that comprises 423 genes, which are enriched in plasma membrane raft organization, cell adhesion, and integrin-mediated signaling pathway. To investigate the high priority (key) genes and signatures of unstimulated macrophage, we implement network centrality measures (degree, betweenness, connectivity, shortest path, cluster coefficient, stress, and topological coefficient) to macrophage co-expression network. Given that most of the real-world networks follow scale-free topology, we first investigated the scale-freeness of the co-expression network and randomly generated a network consisting of co-expression network nodes (Fig. 3b, Supplementary Table S2). Interestingly, we observed that the co-expression network follows scale-free topology based on the power-law distribution (*r*^2^ = 0.92) with only 7.86% (657) of nodes have a high degree (Hub^50^) representing the scale-free property of the co-expression network. Hub^50^ genes are significantly enriched in cell cycle, cytokine, mediated signaling pathway, interferon-alpha response, type 1 interferon response, DNA replication, defense response to virus, and interferon-beta response pathways (− log10(*P*) <  − 6). Furthermore, we calculated additional network centralities distribution and correlated with degree centrality. The relationship between connectivity and degree of nodes shows a significant positive correlation (*r*^2^ = 0.82) suggesting the Hub^50^ nodes and their modules are highly connected (Fig. 3c) while degree and betweenness centrality are not significantly correlated (*r*^2^ = 0.12) (Fig. S1b).

Since a few crucial nodes exhibit increased connectivity and/or central to a network, we concentrated our analysis on TFs enrichment in significant modules and centralities. Towards this, we explored the TFs distribution among co-expressed modules^48^. We demonstrated that the turquoise module has a maximum (22) TFs followed by purple (11), lightcyan (10), and yellow (10) than any other module (Fig. 3d, Supplementary Table S2). Additionally, to ascertain the centrality enrichment significance of TFs, we calculated the average centralities of TFs and genes in co-expression network^48^. We demonstrated that average centralities; average connectivity (96.09), average betweenness (0.020), and average stress (57,764,373.43) of TFs are significantly higher than genes within co-expression network (Wilcoxon matched pairs signed rank test, *P* < 0.0001 for all comparisons). Consistent with these results, we also discovered that the average shortest path is significantly shorter for TFs (6.84) in the co-expression network (Supplementary Fig. S1c–h, Fig. 3e, Supplementary Table S2). Additionally, we report that some of the genes with high connectivity are involved in DNA replication, cell cycle, viral gene expression, DNA repair, cytoplasmic translation, and post-replication repair biological processes. Whereas, high connectivity TFs are enriched in white fat cell differentiation, adipogenesis, IL-9 signaling, nuclear receptors in lipid metabolism and toxicity signaling pathways. Furthermore, we found that genes form bicluster1 are significantly enriched as hub^50^ genes demonstrating their importance in maintaining homeostasis across five diverse mice strains (Hypergeometric test, *P* < 1.4e-29) (Fig. 3f, Supplementary Table S2). Taken together, our analyses describe that TFs are assigned to some of the largest modules and possess significantly high topological centralities in co-expression network^32,48^.

### Integrative systems biology identified differential chromatin accessible regions in unstimulated macrophage of diverse murine strains

**Figure 4:**
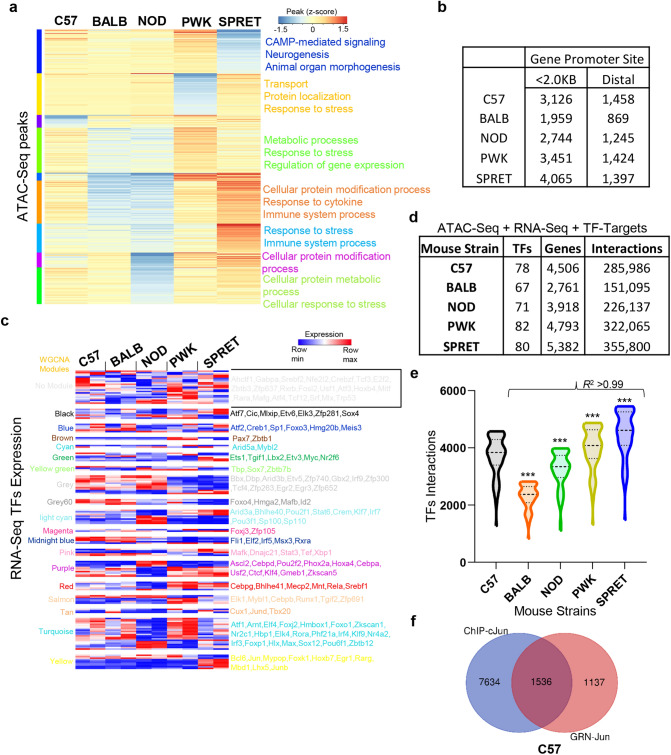
Differential chromatin accessibility and Transcription factors (TFs) interaction in unstimulated macrophage to maintain basal homeostasis in diverse murine strains. (**a**) Heatmap of accessible chromatin peak genes clustered by k-mean clustering and annotated with enriched biological processes in five mice strains. (**b**) Distribution of peaks in the promoter and distal region in five murine strains. (**c**) Heatmap of 124 Transcription Factors (TFs) was expressed across five strains of mice in RNA-seq (TPM > 1). The TFs are grouped according to their WGCNA assigned modules. The clustered columns represent the expression in mice strains, whereas each row represents a TF. Data is row-mean normalized and row clustered using correlation distance and average linkage. (**d**) Gene regulatory network (GRN) components of expressed TFs, expressed and chromatin accessible genes (TPM > 10) in C57,BALB, NOD, PWK, and SPRET murine strains. (**e**) Network analysis of individual GRNs revealed differential interactions for the same TF in all five mice strains at unstimulated macrophage (correlation (*r*^2^ = 0.99) and *P* < 0.0001 when compared to C57 strain). (**f**) ChIP-Seq of cJun and interactions of Jun in GRN of C57 murine model identified more than 57% of target genes correctly.

To investigate the regulatory relationships between TFs and genes in resting macrophage of understudied mice strains, we modeled the Gene Regulatory Network (GRN) by unraveling the ATAC-seq dataset provided by Link et al.^4^. We found substantial distinctions in gene-specific open chromatin regions of five murine strains at resting macrophage (*P* < 0.05, Fig. 4a). Subsequently, the open chromatin regions were integrated with RNA-seq to find expressed TFs and their target genes (Fig. 4b)^49^. This lets us identify 124 expressed TFs across understudied strains in RNA-seq (TPM > 10) (Fig. 4c, Supplementary Table S3). However, most of the TFs express consistently among strains, some TFs express more in certain murine strain. For example, Irf7, Foxp1, Pou2f2 in NOD, and Stat3, Stat6 in SPRET. Interestingly, most of C57 and BALB strain TFs display similar expression behavior, except some TFs (Hmga2, Actf2) express more in C57 than BALB. Afterward, to model the murine strain-specific regulatory relationships, we retrieved prior TF-target relationships from Pscan database^50^ and integrated ATAC-seq as well as RNA-seq datasets^12,48^. As a result, we build five understudied strains-specific resting/unstimulated macrophage GRNs with a distinct number of nodes and edges (Fig. 4d, Supplementary Table S3). Intriguingly, GRNs encompasses 78, 68, 71, 82, and 80 TFs along with 4506, 2760, 3918, 4793, and 5382 target genes in C57, BALB, NOD, PWK, and SPRET strains, respectively. We reported that BALB has the smallest GRN comprising 2828 nodes with 151,095 edges, while SPRET has the largest GRN encompassing 5462 with 355,800 edges. To identify the functional significance of TFs in GRNs, we categorized the TFs based on target interactions. Our analysis found that TFs with maximum interactions are involved in cancer, hepatitis B and C, acute myeloid leukemia, and human T-cell leukemia virus 1 infection, mitophagy, cytosolic DNA-sensing pathway, and parathyroid hormone activity. In contrast, TFs with minimum interactions are involved in viral carcinogenesis, TNF signaling, IL-17 signaling pathway, and Toll-like receptor signaling pathways.

Subsequently, to categorize the interaction behavior of a TF in GRNs, we focused on network analysis of five individual GRNs. As a result, we showed different interactions for the same TFs in all five murine strains (Fig. 4e, Supplementary Table S3). Some of these TFs (Bcl6, Zfp281, Rxra, Tcf3, Klf4, Egr2, Rela, and Zfp740) with maximum interactions are involved in growth, development, homeostasis, and immunity^51^. Additionally, we uncovered that interactions for the same TF are higher in PWK and SPRET than C57 and lower in BALB and NOD suggesting the complex dynamics of unstimulated macrophage to maintaining basal homeostasis. For example, Stat3 TF has 3985, 2469, 3467, 4232, and 4727 target gene interactions in C57, BALB, NOD, PWK, and SPRET murine strains, respectively. Similarly, Stat6 TF has 3930, 2436, 3414, 4173, and 4667 target gene interactions in C57, BALB, NOD, PWK, and SPRET murine strains, respectively. Likewise, we also showed that Jun TF exhibits 2700, 1694, 2372, 2891, and 3222 target gene interactions in C57, BALB, NOD, PWK, and SPRET murine strains, respectively. Further, to validate our candidate GRNs, we compared cJun ChIP-Seq peaks in C57 and validate that our integrative approach identified more than 57% of target genes correctly (Fig. 4f). Collectively, these differences in the TFs expression, promoter occupancy, and interactions within GRNs, suggest divergence in the unstimulated macrophage as the determinant of the organismal plasticity to maintain the intricacy of basal homeostasis gene regulation.

### Modeling of resting macrophage GRN atlas identified immune system enrichment

**Figure 5:**
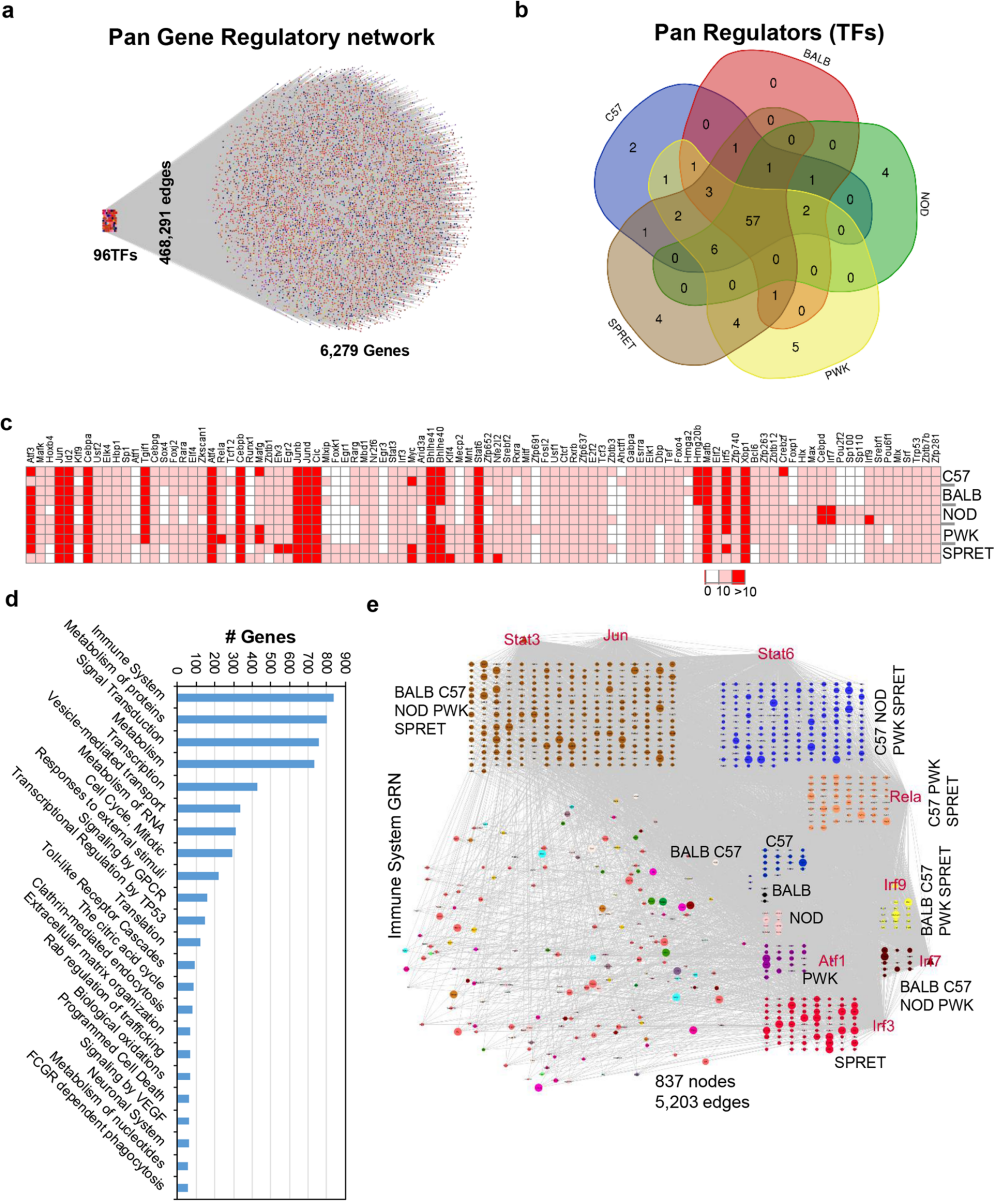
The five consequential GRNs modeled pan basal regulation atlas of unstimulated macrophage in the murine model. (**a**) The pan GRN with 6375 nodes (6279 genes and 96 TFs) and 468,291 edges (interactions) across five mice strains. (**b**) Venn diagram representing the expression of unique and shared 96 TFs in five strains of mice. 57 TFs are expressed in all strains. (**c**) Heatmap of 96 Transcription Factors (TFs) present in pan GRN expressed across five strains of mice. The clustered columns represent TF expression, whereas each row represents different mice strain using correlation distance and average linkage. (Red: TPM > 10, White: TPM < 10). (**d**) Gene and pathway ontology identification of pan GRNs nodes by ClueGO. Some of the significantly enriched ontology terms are immune system, protein metabolism, signal transduction, transport, cell cycle, GPCR signaling, and RNA Metabolism (*P* ≤ 0.001) (Supplementary Figure S2). (**e**) Pan immune system GRN (5203 edges with 837 nodes). There are eight TFs (Stat3, Jun, Stat6, Rela, Irf9, Irf7, Irf3, and Atf1) involved in pan immune GRN. The bigger size of the node denotes Hub^50^ genes from the co-expression network analysis.

Given that five murine strains under study exhibit most of the genetic diversity associated with the human population, a resting/unstimulated macrophage GRN atlas will serve as a benchmark for overarching regulatory repertoire^35,49,52^. To understand the overall (pan) basal homeostasis regulation in resting macrophage, we merged all five-strain specific GRNs interactions and identified the comprehensive edge-based interactions. The resulted pan GRN possesses 6375 nodes (6279 genes and 96 TFs) with 468,291 edges across five mice strains (Fig. 5a, Supplementary Table S4). Interestingly, among the discovered TFs, most of the TFs (57) are conserved among five strains and enriched in SMAD2/3 nuclear pathway, IL-6 signaling pathway, AP-1 transcription factor network, IL-5 regulation of apoptosis, FRA pathway, p38 α/β MAPK downstream pathway, adipogenesis, IL-4 signaling pathway, IL-2 signaling pathway, and transcriptional regulation of white adipocyte differentiation (Fig. 5b). Additionally, to detect the TFs expression difference among strains, we explored the RNA-seq expression analysis. As a result, we identified that 96 TFs expressed differentially across five strains of mice (TPM > 10) (Fig. 5c, Supplementary Table S4). Furthermore, to understand the biological significance of genes and TFs, we performed gene and pathway ontology analysis of pan GRN. Intriguingly, the analysis revealed that immune system, protein metabolism, signal transduction, transcription, transport, cell cycle, response to external stimuli, GPCR signaling, and RNA Metabolism are significantly enriched ontology terms (*P* ≤ 0.001) (Supplementary Fig. S2, Fig. 5d, Supplementary Table S4). These ontology terms are some of the well-studied pathways regulated by LDTF controlled SDTFs of macrophage particularly in maintaining cellular homeostasis, and immune response to stimuli. Consequently, the BMDM macrophage influence by endogenous factors; metabolism, homeostatic regulatory signals, and systemic factors will ultimately determine their basal function.

Additionally, we extracted the genes/TFs involved in the pan immune system. Noticeably, we reported that pan immune system GRN (837 nodes with 5203 edges) comprise eight TFs (Stat3, Jun, Stat6, Rela, Irf9, Irf7, Irf3, and Atf1) interacting with 829 genes across five mice strains (Fig. 5e, Supplementary Table S4). Remarkably, we found that Stat3 and Jun participate in all five mouse strains GRNs, while Atf1 and Irf3 contribute to PWK and SPRET GRN, respectively. Additionally, we discovered that Stat6 is not part of BALB GRN, Rela is not involved in BALB and NOD GRN, Irf9 and Irf7 are not an element of C57 and SPRET GRNs, respectively. Nevertheless, it is well established that these TFs play a vital role in the regulation of the immune system and interferon responses^37,38,53–56^. Furthermore, the network centrality measure of immune system GRN components will highlight the most connected nodes in the co-expression network. The analysis identified that 55hub^50^ genes from BMDM associated gene co-expression networks are part of pan immune GRN signifying its central regulation. Cooperatively, the modeling of resting macrophage GRN atlas, the identification of immune system TFs and genes will provide an excellent resource to the scientific community to explore the genetic disparities to maintain basal homeostasis and immunity across different genetic variants.

### Conserved immune system and signal transduction GRNs across five murine strains

**Figure 6:**
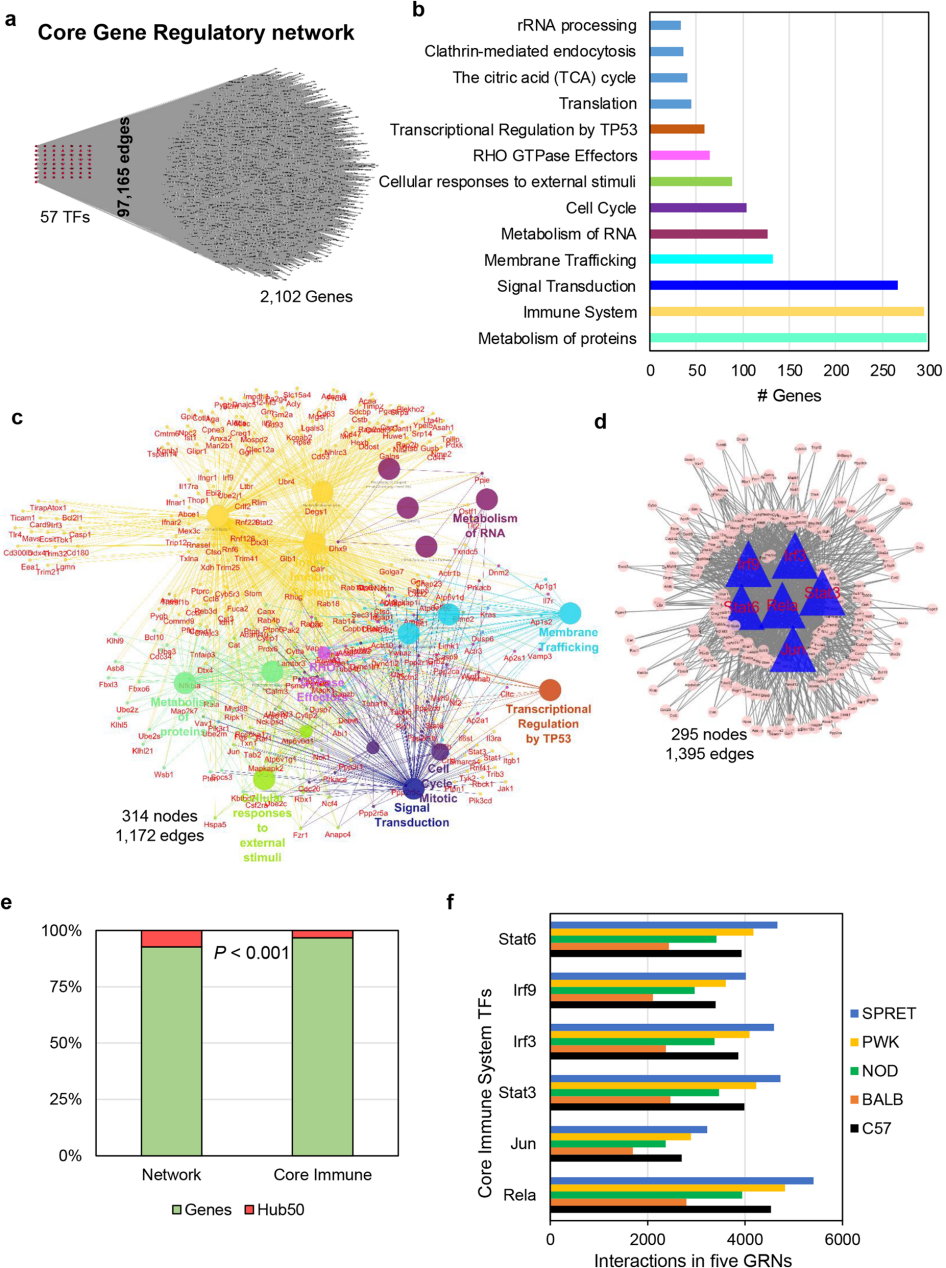
Immune system and signal transduction GRNs are statistically conserved across unstimulated macrophage of five diverse murine strains. (**a**) The merger of five consequential GRNs predicts the core GRN (97,165 edges with 2159 nodes) conserved across five strains of mice. The core GRN has 57 TFs and 2102 genes. (**b**) Gene and pathway ontology of core GRN nodes by ClueGO revealed some of the significantly enriched ontology terms; protein metabolism, immune system, signal transduction, membrane trafficking, RNA metabolism, cell cycle, cellular responses to external stimuli, RHO GTPase effectors, and transcriptional regulation by TP53 (*P* ≤ 0.001) (Supplementary Figure S3). (**c**) The immune system ontology gene network is shared with other significant ontologies. Signal transduction and membrane trafficking have the most common genes involved with the immune system. (**d**) Core immune system GRN (1395 edges with 295 nodes). There are six TFs (Stat3, Jun, Stat6, Rela, Irf9, and Irf3) involved in core immune GRN. (**e**) Core immune genes are significantly enriched in Hub^50^ (Hypergeometric enrichment test, *P* < 0.001) representing their importance in the robustness of the co-expression network. (**f**) The interactions of the core GRN immune system TFs in five strain-specific GRNs.

The conceptual shared genetic regulation between five genetically diverse murine strains will describe the mutual GRN for every individual irrespective of the human race. To study the conserved GRN across murine genetic diversity^4^, we assimilated all five consequential GRNs and identified the core GRN. As a result, we retrieved 2159 nodes (57 TFs) connected through 97,165 edges, conserved across understudied murine strains (Fig. 6a, Supplementary Table S5). Though 57 TFs contribute to core GRN, the number of target gene connections are different in the network. For example, 12 TFs (Klf4, Zfp281, Egr2, Rela, Zfp740, Mafb, Ctcf, Bhlhe40, Sp1, Zfp263, Nr2f6, and Zbtb7b) have more than 2000 target connections, while 45 TFs have less than 2000 target connections. To understand the biological significance of core GRN, we performed gene and pathway ontology by ClueGO. The analysis revealed some of the significantly enriched ontology terms are protein metabolism, immune system, signal transduction, membrane trafficking, RNA metabolism, cell cycle, cellular responses to external stimuli, RHO GTPase effectors, and transcriptional regulation by TP53 (*P* ≤ 0.001) (Supplementary Fig. S3, Fig. 6b, Supplementary Table S5). Additionally, to investigate the core GRN genes involved in enriched pathways we extracted the associated and shared genes between significant ontologies. Remarkably, we report that signal transduction and membrane trafficking have the most common genes participating with the immune system (Fig. 6c, Supplementary Table S5).

Furthermore, to identify the regulators governing the immune system, we extracted the core immune system GRN. Consequently, we identified six TFs (Stat3, Jun, Stat6, Rela, Irf9, and Irf3) involved in core immune GRN (295 nodes with 1395 edges) with Jun possess < 200 target connection than the other five TFs. (Fig. 6d, Supplementary Table S5). Excitingly, these TFs play a crucial role in cell stress, immunodeficiency autoimmunity, NF-κB activation, interferon response, and cancer^53–55^. Additionally, network analysis of core immune GRN nodes in the co-expression network provides a significance in all five murine strains. Interestingly, we uncovered that 10 Hub^50^ genes (Stat1, Nck1, Cdc20, Ube2c, Cyb5r3, Csf2ra, Dctn2, Tubb5, Crk, and Stat2) from the BMDM-associated co-expression network are part of core immune GRN signifying their predominant regulation (Hypergeometric test, *P* < 0.001, Fig. 6e, Supplementary Table S5). Furthermore, to identify the overall core immune TFs regulation through five understudied murine strains, we calculated the total target interaction of six TFs (Stat3, Jun, Stat6, Rela, Irf9, and Irf3) in each GRN. The comparative interaction analysis revealed that NOD and BALB specific GRNs have fewer target interactions for six TFs than C57, while PWK and SPRET have more interactions for six TFs than C57 displaying the disparity in the influence of TFs at resting macrophages in different strains (Fig. 6f, Supplementary Table S5). For example, Irf3 TF has 3862, 2374, 3373, 4091, and 4592 target gene interactions in C57, BALB, NOD, PWK, and SPRET murine strains, respectively. Similarly, Irf9 TF has 3397, 2108, 2963, 3605, and 4016 target gene interactions in C57, BALB, NOD, PWK, and SPRET murine strains, respectively. While, Rela TF has 4531, 2795, 3943, 4821, and 5407 target gene interactions in C57, BALB, NOD, PWK, and SPRET murine strains, respectively. Collectively, this analysis provides the conserved GRN governing the basic immune functions and their robustness through the co-expression network in five murine strains.

### Distinct TFs and GRNs of unstimulated macrophages in five murine strains to maintain basal homeostasis

**Figure 7:**
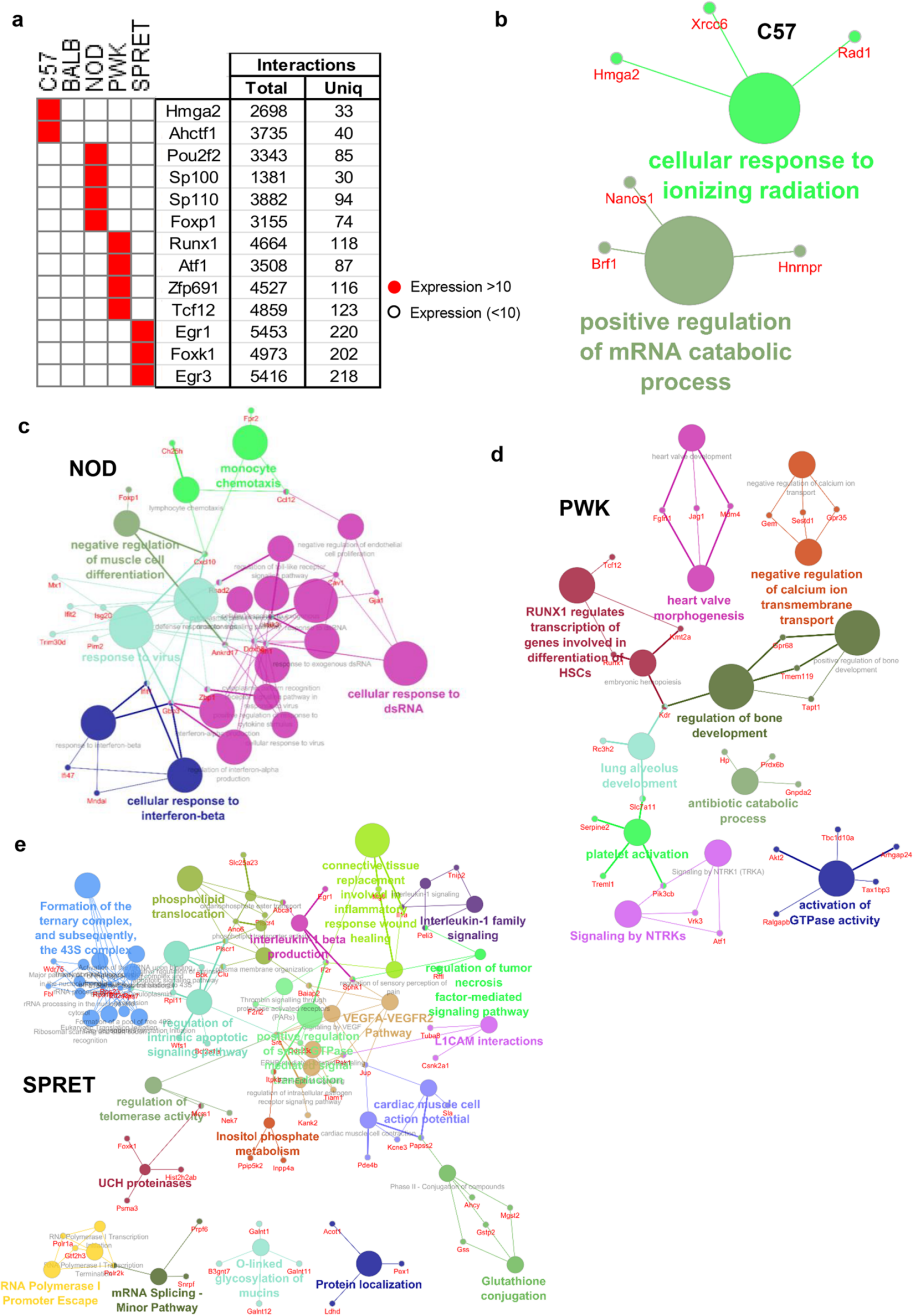
Distinct TFs and GRNs in unstimulated macrophage of diverse genetically defined murine strains to maintain basal homeostasis. (**a**) Heatmap of 13 TFs expressed individually in each strain of mice (Average TPM ≥ 10). Total and unique interactions (genes with average TPM ≥ 10) are mentioned correspondingly. (**b**) Hgma2 and Ahctf1 specific C57 GRN (47 nodes, 72 edges). This GRN is involved in DNA damage and cell cycle. (**c**) Pou2f2, Sp100, Sp110, and Foxp1 specific NOD GRN (93 nodes, 273 edges). This GRN is involved in negative regulation of muscle cell differentiation, interferon signaling and mature B cell differentiation. (**d**) Runx1, Atf1, Zfp691, and Tcf12 specific PWK GRN (112 nodes, 434 edges). This GRN is involved in RUNX1 regulation of HSCs differentiation, signaling by NTRKs and transcription. (**e**) Egr1, Foxk1, and Egr3 specific SPRET GRN (221 nodes, 637 edges). This GRN is involved in interleukin-1 beta production, UCH proteinases and cell migration. There is no TF expressed only in BALB mice. Triangles are TFs and circles are expressed chromatin accessible genes. The gene and pathway ontology of GRNs nodes was done by ClueGO (*P* < 0.05).

Despite basal homeostasis and immunity being conserved across all understudied murine strains for unstimulated/resting macrophages, there are some differences, which exist as far as TFs-target regulation is concerned. To investigate these distinct TFs-target relationships separately, we retrieved TFs and genes which are expressed individually in each murine strains (average TPM ≥ 10), have chromatin accessible and display TF-target relationships from Pscan database. Interestingly, we identified 13 TFs expressed individually in each murine strain (Fig. 7a, Supplementary Table S6). The pathway analysis revealed that these TFs are involved in DNA damage, cell cycle, mature B cell differentiation, interferon signaling, pluripotent stem cells regulation, differentiation of HSCs, cancer/ aldosterone synthesis, generic transcription pathway, myogenesis/ differentiation of HSCs, response to stress, cellular glucose homeostasis, and cell migration (Supplementary Fig. S4a, Supplementary Table S6). Furthermore, we retrieved total and unique target interactions per TF in each strain and predicted the functional annotation by ClueGO. Remarkably, we report two TFs (Hgma2 and Ahctf1) unique to C57 murine strains with C57 specific GRN (47 nodes, 72 edges) enriched in response to ionizing radiation^57^ and mRNA catabolic processes^58^ (Supplementary Fig. S4b, Fig. 7b, Supplementary Table S6). Correspondingly, we account four TFs (Pou2f2, Sp100, Sp110, and Foxp1) unique to NOD murine strains with NOD specific GRN (93 nodes, 273 edges) enriched in negative regulation of muscle cell differentiation^59^, interferon-beta response, response to the virus, dsRNA response and monocyte chemotaxis^60,61^ (Supplementary Fig. S4c, Fig. 7c, Supplementary Table S6). Likewise, we found four TFs (Runx1, Atf1, Zfp691, and Tcf12) unique to PWK murine strain with PWK specific GRN (112 nodes, 434 edges) enriched in RUNX1 regulation of HSCs differentiation^62^, signaling by NTRKs^63^, antibiotic catabolic process, activation of GTPase, and transcription (Supplementary Fig. S4d, Fig. 7d, Supplementary Table S6). Consistently, we identify three TFs (Egr1, Egr3, and Foxk1) unique to SPRET murine strain with SPRET specific GRN (221 nodes, 637 edges) enriched in interleukin-1 beta production, wound healing, UCH proteinases, telomerase activity, and cell migration^64,65^ (Supplementary Fig. S4e, Fig. 7e, Supplementary Table S6). Strikingly, we noticed that there is no TF expressed only in the BALB murine model because the expression pattern of TF/genes is comparable to the C57 murine model. Taken together, these observations highlight the disparities in GRNs resulting in phenotypic differences of unstimulated macrophage for understudied murine strains.

### The human population-based comparative analysis discovered expression inequalities in immunity-related genes and TFs regulating homeostasis in unstimulated macrophages

**Figure 8:**
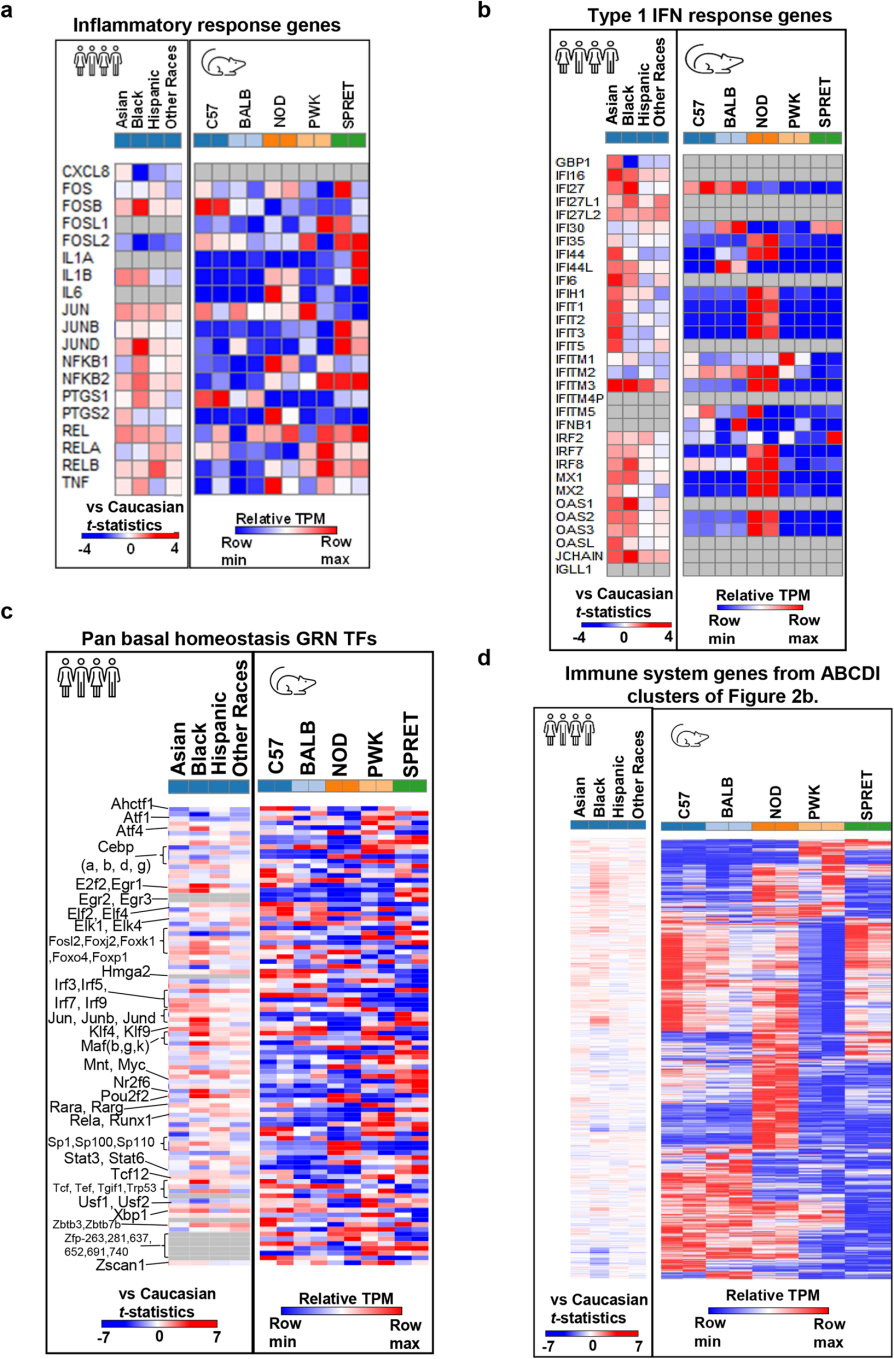
Transcriptome based comparative analysis identified expression disparities between the human population and five murine strains. (**a**) Heatmap of 19 inflammatory response genes expression in the different human populations (Asian, Black, Hispanic, and other races with regard to (w.r.t.) Caucasian individuals, and unstimulated macrophages of five murine strains (C57, BALB, NOD, PWK, and SPRET). (**b**) Heatmap of 32 type 1 IFN response genes expression in different human populations and unstimulated macrophages of five murine strains. (**c**) Heatmap of 96 pan basal homeostasis GRN TFs expression in different human populations and unstimulated macrophages of five murine strains. (**d**) Heatmap of 730 immune-related genes (form cluster A, B, C, d, and I of Fig. 2b) expression in different human population and unstimulated macrophages of five murine strains. Individual heatmap scale for human and murine samples.

The comparative transcriptomic analysis provides a better understanding of translational implication between model systems. Here, we try to understand the expression activity-based relationship of genes/TFs in different biological processes between human and mice systems. A recent population-based transcriptome-wide study^56^ on different human races (Caucasian, Asian, Black, Hispanic, and other races) provides an opportunity for comparative analysis with five understudied murine strains (C57, BALB, NOD, PWK, and SPRET). The RNA-seq datasets for the human population were compared with Caucasian individuals to identify the disparities at the basal/unstimulated level. We aimed to compare the expression of TFs and genes involved in the inflammatory response, type 1 IFN response, pan basal homeostasis GRN, and immune system in different human races and mouse strains. It is worth mentioning that Cole et al., used Caucasian individuals as a reference, thus any gene positively regulated gene in other human races are possibly downregulated in Caucasian population or vice-versa. Interestingly, we uncovered that most of the 19 inflammatory response genes reported by Cole et al*.*, are expressed more in Black and Asian individuals with regard to (w.r.t.) Caucasian individuals, whereas these genes are expressed more in PWK and SPRET murine strains at resting/unstimulated macrophages^56^ (Fig. 8a, Supplementary Table S7). Correspondingly, we found that 32 type 1 IFN response genes reported by Cole et al., have enhanced expression in Asian and Black individuals w.r.t Caucasian individuals, comparably these genes display increased expression in NOD murine strain at unstimulated macrophage^56^ (Fig. 8b, Supplementary Table S7). The existing predisease/unstimulated disparities in inflammation and interferon response highlight that some human races require additional interventions to counter any stress manifestation. Additionally, to test our pan GRN atlas for the basal genetic disparity study, we explored our pan GRN atlas TFs expression in the human population-based transcriptome. Consequently, we discovered that most of TFs exhibit heightened expression in the Black and Hispanic population w.r.t Caucasian individuals (Fig. 8c, Supplementary Table S7). Finally, we also tested the unstimulated/resting macrophages immunity-related genes in understudied murine strains (clusters A, B, C, D, and I from Fig. 2b). As a result, we observed that most of the genes display amplified expression in Black and Asian individuals’ w.r.t Caucasian individuals (Fig. 8d, Supplementary Table S7). In Addition, we illustrate that immunity-related genes are expressed differentially in five murine strains with NOD displaying increased expression for a huge chunk of genes. Collectively, these observations signify the study of strain/race specific GRNs and extensive resources to study basal homeostasis, immunity, disease introduction, and recovery strategies across genetic variations in murine as well as human variations across different races during unstimulated/resting macrophages^4,56^.

## Discussion

Macrophages are important immune cells ubiquitous to almost all body tissues and play significant roles in homeostatic regulation of tissue development, maintenance, repair and remodeling^66^. Although in response to tissue injury, systemic macrophage play key roles, which have extensively been investigated, the importance of resident (unstimulated) macrophage remains relatively poorly defined. It is known that tissue resident macrophage could be influenced by endogenous factors such as metabolism, other homeostatic regulatory signals and/or even factors of systemic origin that may ultimately determine their basal functions. However, in this study, we only focused on the factors associated with murine inter-strain differences as well as differences associated with various human races. These studies demonstrate gene expression changes and their networking influenced by the accessibility of macrophage specific binding sites in transcription-regulatory elements. The rationale behind TF-target gene interactions modeling and characterization is to unravel the comprehensive genome-wide regulatory atlas of an organism irrespective of the exiting genetic variations in different conditions^30,35,37,38,49,51,52,67^. Reconstructing such interactions has revealed that diverse cellular networks are administered by universal principles, and directed to the unrevealing of collective and discrete genetic components and signaling pathways coupled with stress introduction^49,51,52,67^. However, current Gene Regulatory Networks (GRNs) are scattered based on the study type, model organisms and genetic variants used in the study^49,52^. There are gaps in the pan GRN for cumulative and distinct TFs/genes in representing most of the human SNPs^4^. Moreover, these discrepancies in the regulatory repertoire are the missing link between gene and disease associations. To address these challenges and comprehend the mammalian, specifically murine and human complete GRNs repertoire of unstimulated/resting macrophage, we studied the alterations in transcriptome expression and ATAC-seq for chromatin accessible promoter regions across five genetically defined murine strains (C57, BALB, NOD, PWK and SPRET) ^37,52,67^. The selection of these inbred mouse strains is extremely crucial based on the earlier demonstration of the total collection of approximately > 50 million SNPs + InDels of the global genetic variations associated with a difference amongst two individual human beings. In the current study, we constructed an unstimulated BMDM co-expression network displaying features of scale-freeness and amplified network topological centralities for TFs. We modeled five strain-specific GRNs to explore the conserved and distinct regulatory connections in understudied murine strains. The expression of GRNs affect strain-specific macrophage activities in different biological processes including maintaining tissue homeostasis, development, and immunity. The subset of lineage-determining transcription factors (LDTFs) act as master regulators and compete with nucleosomes to bind on the DNA. LDTFs act hierarchically upstream to trigger the signal-dependent transcription factors (SDTFs) during macrophage activity. Furthermore, other epigenetic changes (non-coding) directly perturb the LDTFs binding, henceforth alter the SDTFs regulatory circuits. Despite the differences in expression of the various murine strains and in the human races, there is guaranteed physiological homeostasis due to distinct transcriptional rewiring of LDTFs regulatory circuits. These macrophage-specific regulatory networks are assembled by LDTFs specific promoters and enhancers elements determining their lineage or enforce tissue-limiting properties. Additionally, the differentiation in regulatory circuits determines the plasticity in macrophage associated genetic crosstalk between metabolic pathways and regulation of gene expression. Identification of pan GRN atlas of unstimulated macrophage provided an extraordinary resource to investigate the basal homeostasis, immune system, and signal transduction. In-depth analyses of pan GRN revealed six TFs and some genes involved in immunity are conserved in all five murine strains and significantly more connected and correlated than other genes in co-expressed modules. Additional striking discoveries pertain to the GRN disparity between understudied murine strains and their strain-specific biological significance in inflammation, type 1 IFN response, immune, and homeostasis transcriptional signatures.

We constructed a comprehensive pan GRN atlas encompassing 6279 genes and 96 TFs (Fig. 5a), representing the compounded regulatory repertoire and underline mechanism to maintain basal homeostasis of unstimulated macrophages. The biological pathway analysis identified that the immune system and signal transduction GRNs are statistically enriched and conserved across understudied murine strains. Most of the nodes in the pan regulatory network are enriched in the immune system including eight TFs (Stat3, Jun, Stat6, Rela, Irf9, Irf7, Irf3, and Atf1) interacting with 829 genes across five mice strains. Additionally, six (Stat3, Jun, Stat6, Rela, Irf9, and Irf3) out of eight TFs are conserved in five murine strains with more connections than other genes in co-expressed modules^30,37^. It is well established that significant regulators generally tend to have high network topological properties than other nodes in the network^48,68^. Therefore, highly connected TFs govern a large component of genetic regulation responsible to maintain basal homeostasis at unstimulated macrophages. Strikingly, these TFs play a crucial role in the immune system and interferon response^37,54,55^.

The comparative transcriptomics analyses discovered the expression correlation amongst different human demographics and murine strains. We explored the TFs/genes expression involved in an inflammatory response, immune system, and interferon response of different human demographics^56^. Remarkably, the population-based study identified the disparities in the regulation of the aforementioned functions and compared them with the Caucasian human population. According to Cole et al*.*, if a gene negatively regulated in any other human races, that gene is possibly upregulated in Caucasian population or vice-versa. Interestingly, 19 inflammatory response genes reported by Cole et al., display enhanced expression in Asian and Black human population, while they express more in PWK and SPRET murine strains in unstimulated macrophage^56^. To name a few, FOSB, NFkB1 and JUND genes enriched in cancer^69,70^ and autoimmune disease^71^ are highly expressed in the Black population for humans while their expression is increased in C57, NOD and SPRET murine strains, respectively. Similarly, 32 type 1 IFN response genes display heightened expression in the Asian and Black human population, while they exhibit high expression in NOD murine strain unstimulated macrophage^4,56^. Likewise, well-known interferon signaling regulators; IRF7 and IRF8 display increased expression in Black and Asian populations, while they are expressed more in NOD murine strain^4,56^. Additionally, most of pan GRN atlas TFs (Atf4, Cebpg, E2f2, Egr1, Foxo4, Foxp1, Irf3, Jund, Klf4, Klf9, Mafb, Mafk, Nr2f6, Pou2f2, Rela, Stat6, Tcf, Xbp1, Zbtb3 and Zscan1) display heightened expression in Black and Hispanic population as compared to Asian and other races^4,56^. Functional pathways enriched by these TFs are a cellular response to hormone stimulus, response to radiation, chemokine production, B cell activation, temperature homeostasis, regulation of cytokine production, lymphocyte differentiation, gland development, and response to lipopolysaccharide^72^. Based on these crucial associations, we believe that our GRNs and their significant players can be used as an extensive resource to study homeostasis regulating pathways depicting, immunity, disease introduction, and recovery strategies across genetic variations in murine as well as human races^4–6,51^. However, it remains to be demonstrated if any of these TFs or their interactome may serve as biomarkers for determining susceptibility to infectious and/or autoimmune diseases. It will be of interest to understand their epigenetic regulation and its correlation with disease susceptibility and treatment.

Our final integrative strategy identified distinct TF-target relationships and enriched biological processes in unstimulated/resting macrophages of five genetically defined murine strains. Remarkably, we reported C57 murine strain has two unique TFs (Hgma2 and Ahctf1) and their unique targets are enriched in response to ionizing radiation and mRNA catabolic processes^57,58^. Notably, Hgma2 play a significant role in stress associated cellular senescence and Ahctf1 in mitotic checkpoint during cell cycle interventions^73^. Similarly, NOD murine strains unique TFs (Foxp1, Pou2f2, Sp100, and Sp110) and their unique targets are enriched in negative regulation of muscle cell differentiation, interferon-beta response, and monocyte chemotaxis^59–61^. Therefore, most of interferon response genes are up regulated by Pou2f2 and Sp100 member family nuclear proteins in NOD murine strain. Whereas, PWK murine strain unique TFs (Runx1, Atf1, Zfp691, and Tcf12) and their unique targets are enriched in RUNX1 regulation of HSCs differentiation, signaling by NTRKs, antibiotic catabolic process, activation of GTPase, and transcription^62,63^. Strikingly, Runx1 promotes hematopoietic stem cells growth and inhibits their apoptosis by stimulating transcription of the Myb and Trib2 genes^74,75^. Correspondingly, we identified SPRET murine strain unique TFs (Egr1, Egr3, and Foxk1) and unique targets are enriched in interleukin-1 beta production, wound healing, UCH proteinases, telomerase activity, and cell migration^65,67^. Remarkably, interleukin-1 beta production and interleukin-1 family signaling activation in SPRET highlight more induction of inflammatory response during sepsis^76^. Interestingly, there is no unique TF/gene expressed only in the BALB murine model because the expression pattern of TF/genes is similar to the C57 murine model^4^. This notable conservation and distinctions in TFs and their target gene set to play a significant role in maintaining the immune system, homeostasis and other basal biological functions^66^. They may also be important determinants of susceptibility to various diseases that need to be explored in further detail. Furthermore, differences in the panel of cytokines/chemokines in unstimulated macrophage, only NOD murine strain derived macrophage showed slight polarization towards M1 while PWK/SPRET strains showed tilting towards M2. However further evidence is needed to confirm these observations.

In conclusion, we generated BMDM basal gene co-expression network, integrated transcriptome to regulome for strain-specific GRNs, discovered significant topological and biological regulators and modules, and distinguished unifying and distinct regulatory networks in five murine strains and among human races. Henceforth, our integrative network science approach facilitated the unraveling of intricate and discrete regulatory atlas of unstimulated macrophages to maintain basal homeostasis in five genetically diverse murine strains.

### Limitation of this study

We understand the limitation of this assumption that the expression levels of a given TF may or may not be correlated with its targets due to various layers of gene regulations from mRNA production to the rate of protein synthesis. We would like to highlight a few studies, which employ similar methodology to predict TF-target relationship^20,77,78^. Dam et al., discussed a comprehensive strategy to build predicted disease associated GRN based on gene co-expression networks^20^. Similarly, other study used correlation among expressed genes to identify module regulated by TFs at different time points^77^. We agree and understand that the ideal situation would be to quantify TF protein levels (proteomics) and correlate them with TF transcript levels^78^. A more direct measure, however, is to integrate ChIP-Seq data of all TFs under similar cellular and physiological conditions. Such circumstances may or may not be flawless since the spatiotemporal gene expression may not fully overlap with protein synthesis. Needless to say, that such studies are not prevalent as of now in commonly used mouse strain and not explored in other mouse strains under study. Furthermore, the epigenetic modulation of TF binding was not explored while establishing the murine strain specific GRN. Additional study on integrating (3C/4C/Hi-C data) can reveal the GRN interactions in higher resolution.

## Materials and methods

### RNA-sequencing and differentially expressed genes analysis

The primary data source of this study is five diverse genetically defined murine strains bone marrow-derived macrophage (BMDM) transcriptomics expression data generated by Link et al.^4^, retrieved from NCBI’s Gene Expression Omnibus^79^ with the accession number GSE109965. The study uses five murine strains (C57, BALB, NOD, PWK, and SPRET) to explore the basal homeostasis with two replicates for each strain. The read count value transcripts per million (TPM) dataset was pre-processed (< 10 was filtered out) for expression threshold in each murine strain. The filtered TPM values were log_2_ transformed for each gene in every sample and fold change concerning each murine strains between each pairwise comparison^48^. We performed differentially expressed genes (DEGs) analysis by DESeq2^80^ as described by Link et al.^4^. This log_2_ transformed expression profiles generated with expression parameters; > 2 for up-regulated genes and < -2 for down-regulated genes. To understand the function of the DEGs, the gene ontology (GO) enrichment was performed using Enrichr^81^, ClueGO^82^ and Kyoto Encyclopedia of Genes and Genomes (KEGG)^83^ enrichment analysis was carried out using iDEP^84^.

### Weighted gene co-expression network construction

The availability of high-resolution, large-scale transcriptome datasets enables co-expression network analysis to identify clusters of highly correlated genes that are potentially co-regulated to maintain Immune homeostasis within diverse genetically defined murine strains. Thus, co-expression networks allow identifying a set of genes, which might participate in a common biological process. To determine basal homeostasis associated common gene signatures, we implemented a correlation-based R package; weighted gene co-expression network analysis (WGCNA)^47^ on RNA sequencing TPM count dataset. Specified the complexity of the multi-course dataset, exhausting a hard threshold would result in loosing of information and may affect the sensitivity too^85^. Hence, a soft-threshold power of 18 along with a scale-free model fit index r^2^ > 0.68 was utilized for maximum scale-free topology, preserving high mean connectivity, and rejecting lesser correlations for genes. The module in WGCNA is assigned by a flexible process, permits to affect the least number of features confined in each module by semi-programmed cutting of the dendrogram, and is denoted by a unique color. We created an elementary WGCNA network utilizing flashClust() and cutreeDynamicTree() algorithms^85^, incorporating all cleaned expression values^85^. The module functional analysis is done by Metascape^72^ for FDR < 0.01 through KEGG, Reactome and GO biological processes for all modules simultaneously. The resulting co-expression network with 8357 nodes and 63,130 edges was visualized in cystoscape^86^ and utilized for further network analysis.

### ATAC sequencing peak calling and Gene Regulatory Network (GRN) construction

The primary data source of this study is a reproducible high-resolution diverse genetically defined murine strains ATAC seq data generated by Link et al.^4^, retrieved from NCBI’s Gene Expression Omnibus^79^ with the accession number GSE109965. We performed strain-specific peak calling and differentially bound TF binding site analysis through HOMER^3^ as described by Link et al.^4^. The GRN construction was done by integrating gene expression (TPM ≥ 10) from RNA-seq, open chromatin regions from ATAC-seq and TF-Target interactions of the aforementioned genes/TF from Mouse Pscan database^50^ (http://159.149.160.88/pscan/). Individual GRNs for genetically diverse murine strains were generated. PAN and core GRNs were constructed by merging all GRNs and finding the conserved GRN across five mice strains, respectively.

### Network analyses

Network topology measures^17,28,32^ such as degree, betweenness centrality, connectivity, cluster coefficient, and stress centrality were calculated using NetworkX^87^. The degree of a node is the total number of connections in a network. The highly connected nodes in the network are identified as hubs. While betweenness centrality determines the frequency of a node in facilitating interactions with other nodes through the shortest paths^88^. Both hubs and bottlenecks (high betweenness) have been exploited for significant nodes discovery in diverse intra- and inter-species interactions^89^. Similarly, connectivity determines the resilience of the network through the measure needed for separating a network into multiple subnetworks. While the clustering coefficient determines the highly clustered components by distinguishing the total number of triangles in the network. Whereas, stress centrality determines the aggregation of shortest paths between all node pairs. Additionally, the average centralities of TFs and the rest of the network were calculated and compared. Hub^50^ are the genes with ≥ 50 connections in the co-expression network^48^. The networks were visualized in Cytoscape 3.7.2^86^.

### Statistical analyses

The number of differential expressed genes (DEGs) maintaining basal homeostasis between different mouse strains was calculated with FDR < 0.05. The correlation between overall connectivity and degree of co-expressed genes is significantly positive (*r*^2^ = 0.82). The Wilcoxon matched pairs signed rank test was used to test the significance of TFs and genes based on network centralities. The Hypergeometric enrichment test was used to test the significance of Hub^50^ genes in the co-expression network and Bicluster1 (830 immune genes). The Hypergeometric enrichment test was used to test the significance of Hub^50^ genes in the co-expression network and immune genes in core immune GRN genes. Gene and pathway ontology identification of GRNs nodes by Enrichr, and ClueGO with *P* ≤ 0.05 and 0.001, respectively.

## Supplementary Information

**Supplementary Information.**

**Supplementary Table S1.**

**Supplementary Table S2.**

**Supplementary Table S3.**

**Supplementary Table S4.**

**Supplementary Table S5.**

**Supplementary Table S6.**

**Supplementary Table S7.**

## Data Availability

All datasets used and generated from this study are accessible through Table S files.

## Materials availability

This study did not generate new unique reagents. Requests for materials and communications with the journal should be addressed to M.S.M. (smukhtar@uab.edu).
