# Supplementary material for: Transcriptional circuitry atlas of genetic diverse unstimulated murine and human macrophages define disparity in population-wide innate immunity
Source: Sci Rep. 2021 Apr 1;11:7373. doi: 10.1038/s41598-021-86742-w (PMC8016976; doi:10.1038/s41598-021-86742-w)
Supplement: Supplementary file 1 — Supplementary Information. [file 41598_2021_86742_MOESM1_ESM.pdf]

Transcriptional Circuitry Atlas of Genetic Diverse Unstimulated Murine and Human Macrophages Define Disparity in Population-wide Innate Immunity

Bharat Mishra[1], Mohammad Athar[2*] & M. Shahid Mukhtar[1,3,4*]

Affiliations

[1] Department of Biology, 464 Campbell Hall, 1300 University Boulevard, University of Alabama at Birmingham, Alabama 35294, USA

[2] UAB Research Center of Excellence in Arsenicals, Department of Dermatology, School of Medicine, University of Alabama at Birmingham, Alabama 35294, USA

[3] Nutrition Obesity Research Center, 1675 University Blvd, University of Alabama at Birmingham, Birmingham, AL 35294, USA.

[4] Department of Surgery, 1808 7th Ave S, University of Alabama at Birmingham, Birmingham, AL 35294, USA

*Correspondence

Correspondence and requests for materials should be addressed to M.A. (mohammadathar@uabmc.edu) and M.S.M. (smukhtar@uab.edu)

Running Title: Murine genetic diverse basal homeostasis regulatory atlas

Supplemental Information

Description of Additional Supplemental Files

Supplementary Table S1: Gene expression pattern of unstimulated bone marrow-derived macrophage (BMDM) across diverse murine strains.

1. Total Gene expression in five murine strains
2. *k*-mean Cluster Enrichment of Expressed Genes
3. Bicluster 1 Genes
4. DEGs at Basal/Unstimulated macrophages in different murine strains

Supplementary Table S2: Gene co-expression network construction and analysis of unstimulated macrophage across diverse murine strains

1. BMDM basal homeostasis gene co-expression network
2. BMDM basal homeostasis gene co-expression network node properties
3. Random and BMDM network degree distribution
4. Module TFs and Genes distibution
5. Average centralities enrichment of TFs and Genes/Other
6. Hub50 functional enrichment
7. High connectivity (>129) genes functional enrichment
8. High connectivity (>130) TFs functional enrichment

Supplementary Table S3: GRNs in five diverse murine strains

1. TFs expression in five murine strains
2. C57 GRN
3. BALB GRN
4. NOD GRN
5. PWK GRN
6. SPRET GRN
7. TFs interaction in five individual GRNs
8. GRN size

Supplementary Table S4: Pan basal homeostasis gene regulatory network

1. Pan basal homeostasis GRN
2. TFs presence in strains
3. GO term enrichment of pan GRN
4. Pan immune system GRN
5. Pan immune system GRN nodes

Supplementary Table S5: Core basal homeostasis gene regulatory network

1. Core GRN
2. GO enrichment of core GRN
3. Core immune system GRN
4. Core immune GRN TF interactions

Supplementary Table S6: Distinct TFs and GRNs for unstimulated macrophages across diverse murine strains

1. Average TPM of all and unique TFs
2. C57 unique GRN
3. NOD unique GRN
4. PWK unique GRN
5. SPRET unique GRN

Supplementary Table S7: Comparative transcriptome analysis identified expression disparities between human population and five murine strains

1. Inflammatory response genes
2. Type 1 IFN response genes
3. Pan gene regulatory network TFs
4. Immune system genes from clusters; A, B, C, D, and I of Fig. 1b

Supplemental figures and legends

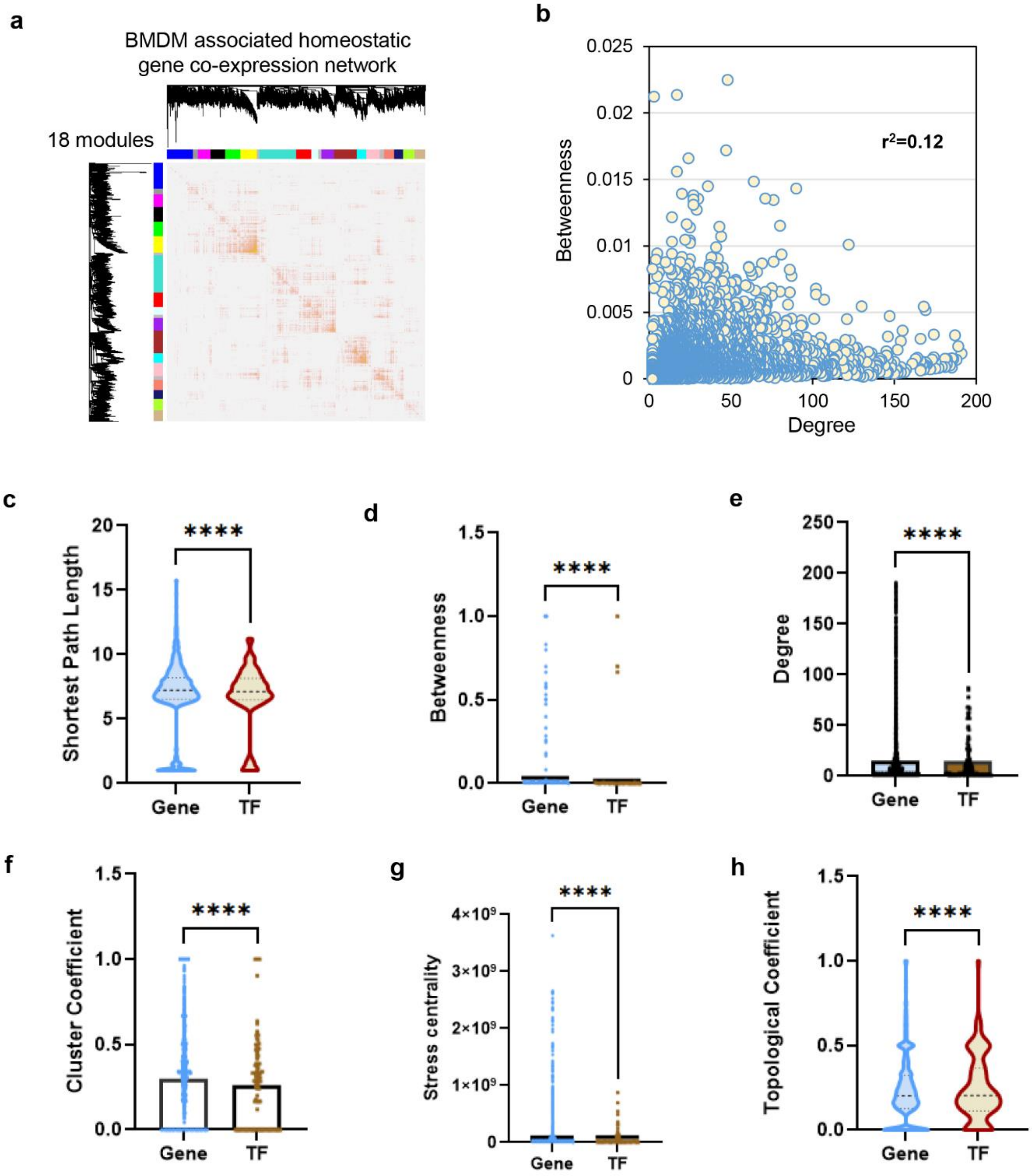

Supplementary Fig. S1. BMDM associated basal homeostatic gene co-expression network analyses. a. Topological overlap matrix (TOM) plot of module assigned genes in five mice strains. **b.** Relationship between degree distribution and betweenness centrality of co-expression network genes. **c - h.** Average centrality (shortest path,

betweenness centrality, degree, cluster coefficient, stress, and topological centrality) of TFs and other genes in BMDM associated homeostatic gene co-expression network (significant<0.05, non-significant (n.s) > 0.05).

Supplementary Fig. S2

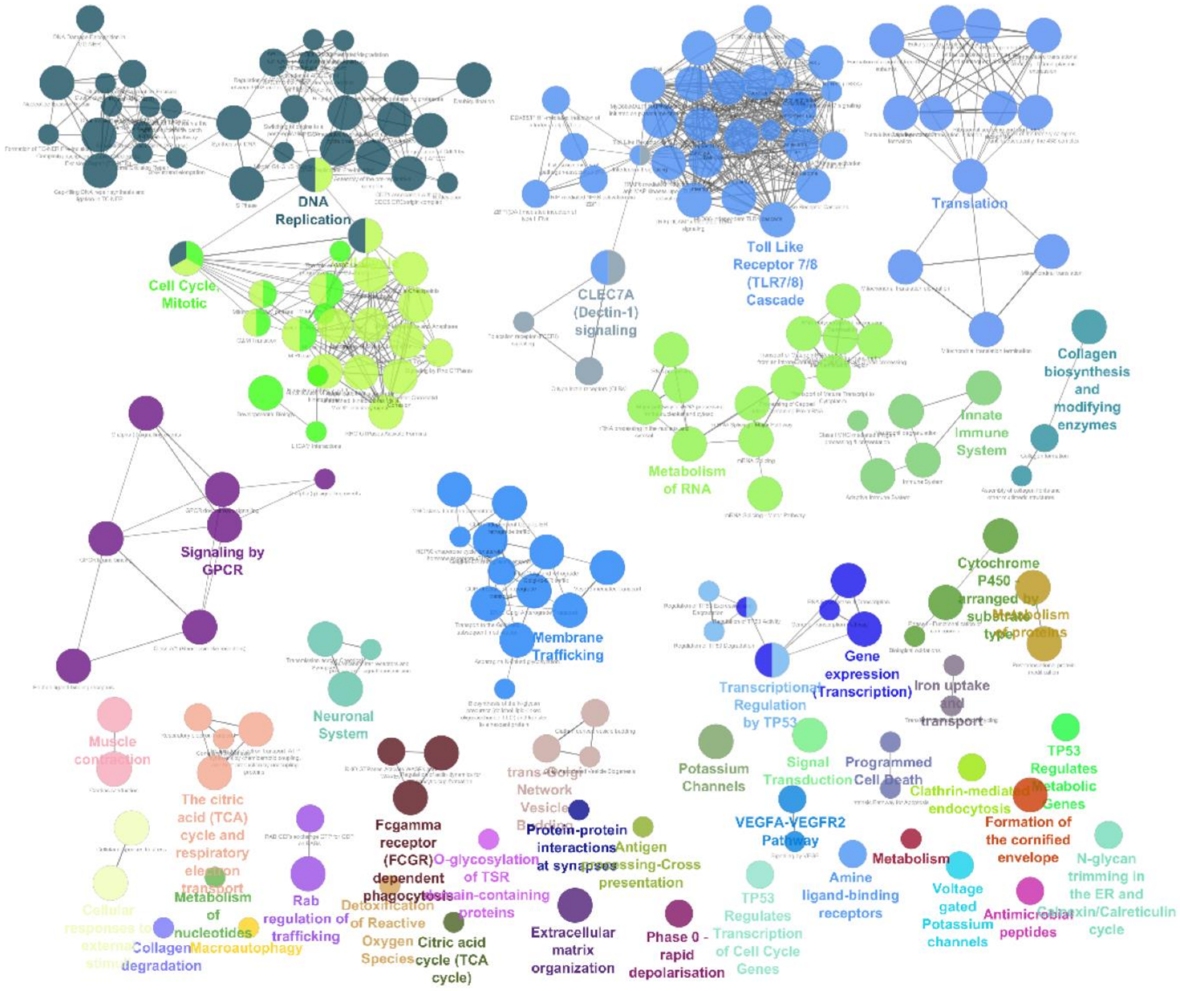

Supplementary Fig. S2. Gene ontology and pathway analysis of pan GRN atlas. Gene Ontology analysis and enrichment representation of pan GRN by ClueGO for *Mus Musculus* [10090] as reference. The parameters set were (minimum number of genes = 3, min percentage = 4.0, GO Fusion = false, GO Group = true, Kappa Score Threshold =

0.4, overview term = smallest *P*-value, and group by kappa statistics = true). Total 196 GO terms were identified with 418 connections among themselves. The final group size after merging was 46. All enriched Reactome pathways representing different colors based on their GO terms. Some of the most abundant enriched GO terms for pathways are Immune System, Metabolism of proteins, Signal Transduction, Metabolism, Post-translational protein modification, Vesicle-mediated transport, Neutrophil degranulation, Membrane Trafficking, Metabolism of RNA, Cell Cycle, Signaling by Rho GTPases, Processing of Capped Intron-Containing Pre-mRNA, RHO GTPase Effectors, Asparagine N-linked glycosylation, and Signaling by GPCR (P<0.001).

Supplementary Fig. S3

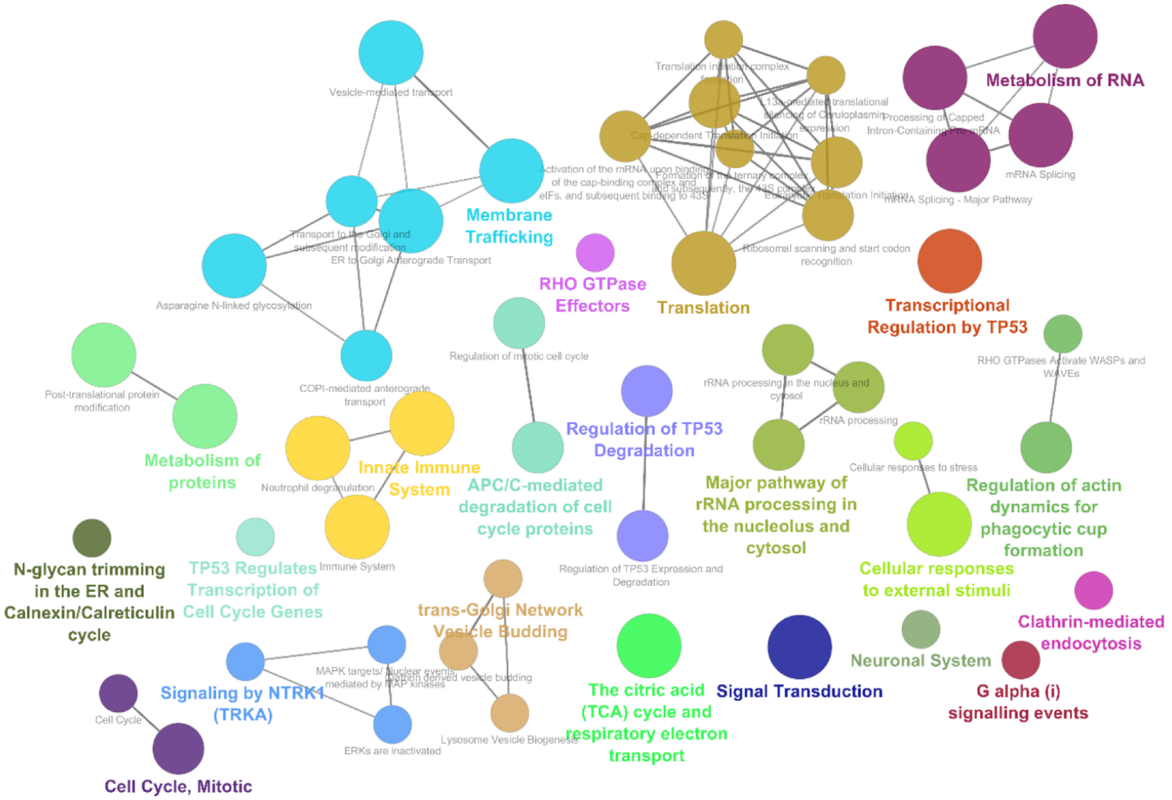

Supplementary Fig. S3. Gene ontology and pathway analysis of core GRN. Gene Ontology analysis and enrichment representation of core GRN by ClueGO for *Mus Musculus* [10090] as reference. The parameters set were (minimum number of genes = 3, min percentage = 4.0, GO Fusion = false, GO Group = true, Kappa Score Threshold = 0.4, overview term = smallest *P*-value, and group by kappa statistics = true). Total 51 GO terms were identified with 49 connections among themselves. The final group size after merging was 22. All enriched Reactome pathways representing different colors based on their GO terms. Some of the most abundant enriched GO terms for pathways are Metabolism of proteins, Immune System, Signal Transduction, Post-translational protein modification, Vesicle-mediated transport, Neutrophil degranulation, Membrane Trafficking, Metabolism of RNA, Cell Cycle, Cellular responses to external stimuli, Asparagine N-linked glycosylation, Cellular responses to stress, Processing of Capped Intron-Containing Pre-mRNA, RHO GTPase Effectors, mRNA Splicing, and Transcriptional Regulation by TP53 ($P<0.001$).

Supplementary Fig. S4

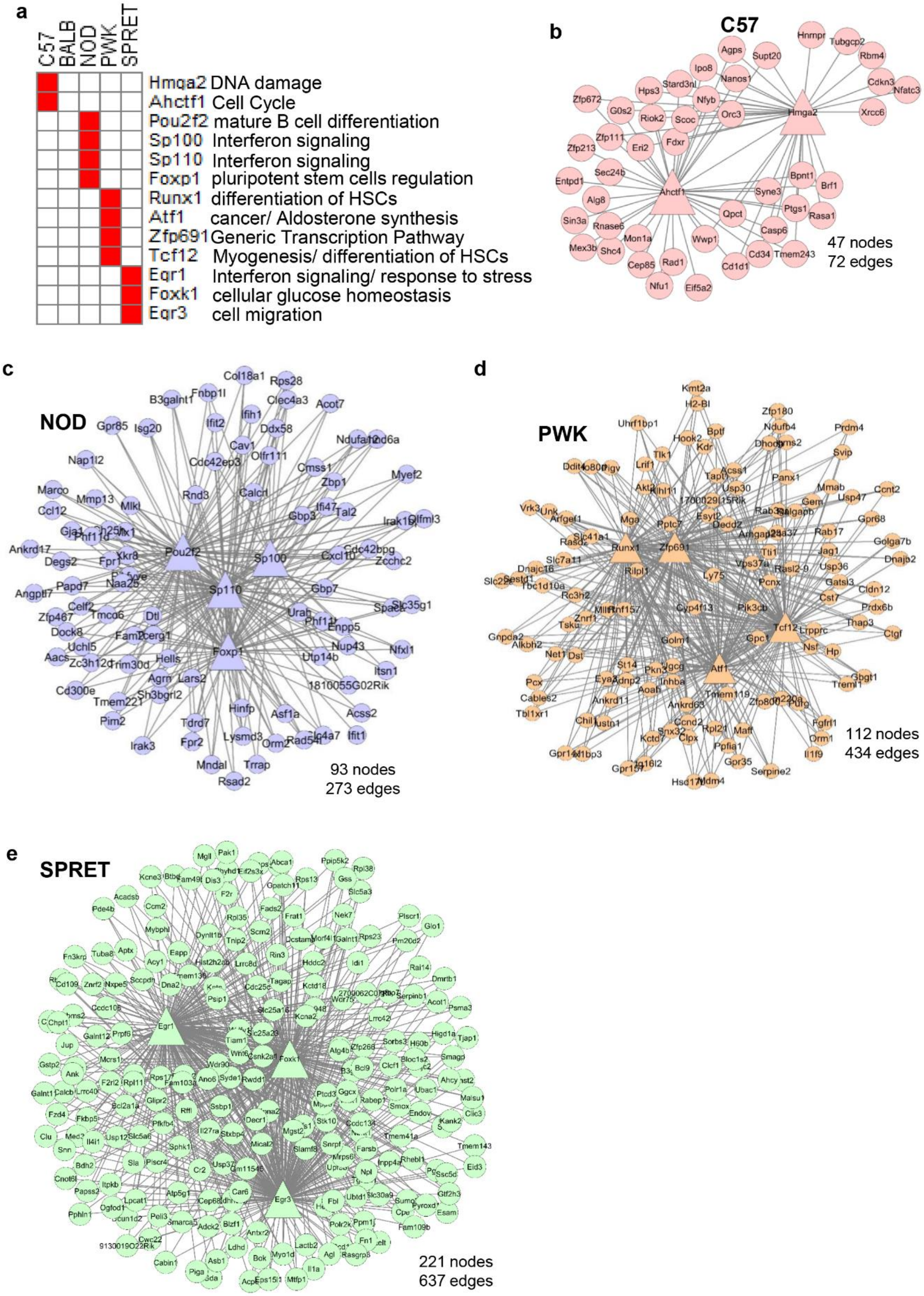

Supplementary Fig. S4. Distinct GRNs in five murine strains. a. Heatmap of 13 TFs expressed individually in each strain of mice (Average TPM≥ 10). Functional annotation is mentioned correspondingly. **b.** Hgma2 and Ahctf1 specific GRN (47 nodes, 72 edges) in C57 mice. **c.** Pou2f2, Sp100, Sp110, and Foxp1 specific GRN (93 nodes, 273 edges) in NOD mice. **d.** Runx1, Atf1, Zfp691, and Tcf12 specific GRN (112 nodes, 434 edges) in PWK mice. **e.** Egr1, Foxk1, and Egr3 specific GRN (221 nodes, 637 edges) in SPRET mice. Triangles are TFs and circles are expressed chromatin accessible genes.
